# Adolescent attachment to parents and peers in singletons and twins born with assisted and natural conception

**DOI:** 10.1093/hropen/hoac012

**Published:** 2022-03-08

**Authors:** M S Flykt, M Prince, M Vänskä, J Lindblom, J Minkkinen, A Tiitinen, P Poikkeus, Z Biringen, R -L Punamäki

**Affiliations:** Department of Psychology and Logopedics, Faculty of Medicine, University of Helsinki, Helsinki, Finland; Department of Psychology, Faculty of Social Sciences, Tampere University, Tampere, Finland; Department of Psychology, Colorado State University, Fort Collins, CO, USA; Department of Psychology, Faculty of Social Sciences, Tampere University, Tampere, Finland; Department of Psychology, Faculty of Social Sciences, Tampere University, Tampere, Finland; Department of Clinical Medicine, University of Turku, Turku, Finland; Department of Psychology, Faculty of Social Sciences, Tampere University, Tampere, Finland; Department of Obstetrics and Gynecology, Faculty of Medicine, University of Helsinki, Helsinki, Finland; Helsinki University Hospital, Helsinki, Finland; Department of Obstetrics and Gynecology, Faculty of Medicine, University of Helsinki, Helsinki, Finland; Helsinki University Hospital, Helsinki, Finland; Department of Human Development and Family Studies, Colorado State University, Fort Collins, CO, USA; Department of Psychology, Faculty of Social Sciences, Tampere University, Tampere, Finland

**Keywords:** adolescence, attachment, twins, singletons, assisted reproductive treatment, ART, infertility, relationship, parents, peers

## Abstract

**STUDY QUESTION:**

Does adolescent attachment to parents and peers differ between singletons and twins born with ART or natural conception (NC)?

**SUMMARY ANSWER:**

Adolescent attachment anxiety with the father was higher among NC singletons than among ART and NC twins, whereas attachment avoidance with the father was higher in ART singletons than in NC singletons and NC twins. No differences were found in attachment to the mother, best friend or romantic partner.

**WHAT IS KNOWN ALREADY:**

Most studies have not found differences between ART and NC singletons in parent–adolescent relationships, but twin relationships may be more at risk. No previous study has examined all four groups in the same study, or specifically looked at attachment relationships.

**STUDY DESIGN, SIZE, DURATION:**

This was an 18-year, prospective and controlled longitudinal study with families of 496 ART singletons, 101 ART twin pairs, 476 NC singletons and 22 NC twin pairs. Families were recruited during the second trimester of pregnancy; the ART group was recruited from five infertility clinics in Finland and the control group was recruited from a hospital outpatient clinic during a routine visit.

**PARTICIPANTS/MATERIALS, SETTING, METHODS:**

Mothers and fathers gave background information for this study during pregnancy, and during the child’s first year and early school age (7–8 years). For the ART group, infertility characteristics and prenatal medical information was also obtained from the patient registry of the infertility clinics. Children (originally 50% girls) filled in electronic questionnaires related to their attachment to mother, father, best friend and romantic partner (*Experiences in Close Relationships—Relationship Structures*) at 17–19 years of age.

**MAIN RESULTS AND THE ROLE OF CHANCE:**

Adolescent attachment anxiety to father was higher in NC singletons than in ART twins, *P* = 0.004 and marginally higher than in NC twins, *P* = 0.06. Adolescent attachment avoidance to father was higher in ART singletons than in NC singletons, *P* = 0.006 and marginally higher than in NC twins, *P* = 0.055.

**LIMITATIONS, REASONS FOR CAUTION:**

The sample size was small especially in the NC twin group and there was drop-out over the 18-year time period, especially among boys and families with lower parental education level. The study only included native Finnish-speaking families. The results could differ in a more diverse population. ART singletons were younger and had fewer siblings than ART twins and NC children, and ART and NC twins had more newborn health risks than ART and NC singletons.

**WIDER IMPLICATIONS OF THE FINDINGS:**

The study adds to a growing body of evidence that neither ART treatments nor being a twin places mother–child relationships or peer relationships at long-term risk. However, in our study, which was the first to examine both ART and twinhood simultaneously, we found that there may be more problems in father–adolescent relationships, but only in ART singletons and only related to attachment avoidance. Our findings suggest that men, as well as women, should receive enough support in pre- and peri-natal health care during and after infertility treatments.

**STUDY FUNDING/COMPETING INTEREST(S):**

This study was funded by Academy of Finland (grant number 2501308988), the Juho Vainio Foundation and the Finnish Cultural Foundation. The authors report no conflict of interest.

**TRIAL REGISTRATION NUMBER:**

N/A.

WHAT DOES THIS MEAN FOR PATIENTS?

This study looks at how singleton and twin adolescents conceived with IVF treatment or naturally differ in their attachment relationships. The attachments refer to the adolescent’s close relationships to their mother, father, best friend and romantic partner. The study followed up on more than 1000 families from pregnancy to the age of 17–19 years. Infertility and its treatments, as well as parenting young twins, can be stressful or cause worry for parenting or child development. However, most studies have not found long-term problems in parent–adolescent relationships in families conceived with IVF. Knowledge is still lacking on parent–adolescent relationships in twins, and about adolescent relationships to friends and romantic partners in those conceived with IVF treatment. Our results show that both singletons and twins conceived with IVF had equally good attachment relationships with their mothers, best friends and romantic partners as naturally conceived adolescents. Attachment relationships to the father were, however, somewhat more problematic, but only among singletons. IVF-conceived singletons showed more emotionally distant attachment relationships with their father than naturally-conceived adolescents. Instead, naturally-conceived singletons showed more anxious, over-dependent attachments with their father than naturally or IVF-conceived twins. Our results thus show that both twins and singletons conceived with IVF treatment generally do well in their later close relationships. Considering the more emotionally distant adolescent attachment to fathers, it might be important that men, as well as women, receive enough support in pre- and peri-natal health care during and after experiencing infertility.

## Introduction

Although assisted reproductive treatments can make the dreams of parenthood come true for infertile couples, the treatments can also be physically and emotionally demanding (Greil *et al.*, 2011). Most research indicates no major long-term risks in well-being and family relations in families conceived with ART (Ilioi and Golombok, 2015; Sydsjö *et al.*, 2015; Vikström *et al.*, 2015). However, some studies do suggest a specific risk for family enmeshment or over-involved parenting (Owen and Golombok, 2009; Cairo *et al.*, 2012; Lindblom *et al.*, 2014). Twin pregnancies are more common among ART than naturally-conceiving (NC) families, and they often involve a higher medical risk. Parents of ART twins experience more parenting stress and mental health problems during the child’s early years as compared to parents of ART singletons, although the extent seems similar to parents of NC twins (Olivennes *et al.*, 2005; Vilska *et al.*, 2009; Tendais and Figueiredo, 2016).

Parent–child relationships may be affected by the stress of sharing resources with two infants. Compared to mothers of singletons, twin mothers have been shown to be less involved, sensitive and communicative with their children (Holditch-Davis *et al.*, 1999; Thorpe *et al.*, 2003; Feldman and Eidelman, 2004), and both mothers and fathers of twins have been found to provide a less structured home environment for their children than parents of singletons (Feldman and Eidelman, 2004). Furthermore, twin parents are found to display more hostility and expectations of conformity to rules (Boivin *et al.*, 2005; Anderson *et al.*, 2015) and report less enjoyable and positive parent–child interactions (Glazebrook *et al.*, 2004; Olivennes *et al.*, 2005). Twins may also be treated less individually (Thorpe *et al.*, 2003), or there may be favoritism (e.g. division between ‘mother’s twin’ and ‘father’s twin’), which may be non-optimal for child development (Thorpe *et al.*, 2003; Trias *et al.*, 2006). However, the above studies documenting parenting problems in twin families have mostly concerned early childhood and it is less clear whether the risk applies to adolescence.

Few studies have examined parent–adolescent relationships in twin or even in singleton ART families. For ART singletons, parent–adolescent relationships have been reported as at least equally warm as among NC singletons (Colpin and Bossaert, 2008; Goisis and Palma, 2021), although one study reported more disciplinary indulgence among ART mothers (Owen and Golombok, 2009). We are aware of only one previous study comparing the family relationships of ART twins and singletons in adolescence. Anderson *et al.* (2016, 2017) found that ART twins had more problematic father–adolescent interactions at 12 years than ART singletons, but the differences disappeared by 17 years of age. No differences were found in mother–adolescent interactions at either time point. We are not aware of any previous studies comparing parent–adolescent relationships in ART and NC singletons and twins to each other, and the previous twin studies also lack the adolescent’s own perspective on relationships.

One of the most crucial aspects of parent–child relationships is attachment: the close affectional bond children develop toward their caregivers (usually the parents) starting from the first year of life (Bowlby, 1969). The evolutionary purpose of attachment is seeking protection from the caregiver under stressful situations (Bowlby, 1969). For young children, this implies physical proximity-seeking, whereas for adolescents and adults, attachments provide a psychological source of safety: that one’s distresses can safely be shared with supportive others. Attachment bond is also accompanied by mental models of the self and others that are based on the quality of parental caregiving but also generalize into other close relationships (Main *et al.*, 1985). When parents are sensitive in perceiving and responding to their children’s needs, children develop secure attachments, enabling them to rely both on themselves and on other people’s support and to express their needs openly (Ainsworth *et al.*, 1978). Instead, children tend to develop insecure attachments when parents are unavailable and unresponsive to child needs (avoidant attachment) or when parents are highly inconsistent in their availability (anxious attachment), leading to concerns about rejection or consistent availability of loved ones in these relationships (Ainsworth *et al.*, 1978). Attachment security is known to be highly beneficial for the socioemotional development of individuals, such as the ability to regulate one’s emotions and form close relationships, with underlying neural-hormonal physiological changes explaining these differences (Beckes *et al.*, 2015; Groh *et al.*, 2017). Early secure attachments also enhance later-life close relationship quality, such as friendships and romantic relations, as secure attachments facilitate reciprocal, supportive relationships between partners (Fraley and Shaver, 2000; Schneider *et al.*, 2001). Individual differences in attachment security and insecurity in adolescence have been conceptualized along two dimensions: Attachment avoidance and attachment anxiety (Bartholomew and Horowitz, 1991; Fraley *et al.*, 2011). Attachment avoidance implies the extent to which individuals shut off their needs for human connection and are uncomfortable with relying on others or having others depend on them (Fraley *et al.*, 2011). Attachment anxiety indicates how much the individual worries about the consistent availability and responsiveness of others in their close relationships and is overly dependent on others. Persons with secure attachment are low on both attachment avoidance and anxiety.

To our knowledge, only one previous study has looked at attachment in ART-conceived children, showing no differences in the attachment patterns between ART and NC singletons toward their mother at 1 year of age (Gibson *et al.*, 2000). Further studies concerning NC twins have only examined twin attachment concordance in adolescence and adulthood (Torgersen *et al.*, 2007; Picardi *et al.*, 2011; Fearon *et al.*, 2014). However, to our knowledge, no previous studies exist on attachment in ART twins, or even in ART singletons, beyond infancy.

In adolescence, the scope of attachment relationships gets broadened and individuals form attachment not only to parents, but also to peers (defined here as best friend and romantic partner) (Hazan and Shaver, 1994; Kobak *et al.*, 2007). Although attachments to parents still remain of primary importance, peer attachments can also impact adolescent well-being and are thus important to consider in research (Flykt *et al.*, 2021).

Research comparing peer relationships in ART and NC twins is lacking, but Golombok and colleagues (2009) showed that at 18 years of age, ART singletons actually reported more confidence in peer relationships as compared to NC singletons. Studies are also scarce and contradictory concerning peer relationships in NC twins. DiLalla (2006) showed that in toddlerhood, twins were observed to be less prosocial with other children than singletons, and at 10–15 years of age, parents of twins rated them as more aggressive than parents of singleton children. However, in another study (Pulkkinen *et al.*, 2003), twins were found to be more socially adaptive than singletons in early adolescence. Although studies comparing romantic relationships between singletons and twins are almost completely lacking, early evidence suggests that singletons may form closer attachments to their romantic partner than twins due to closeness inherent in the twin relationship itself (Tancredy and Fraley, 2006; Schwarz *et al.*, 2015).

To capture the independent, long-term role of both ART treatments and being a twin in the development of adolescent’s multiple attachment relationships, this study examined whether ART and NC twins and singletons differ from each other in their attachment avoidance and attachment anxiety toward parents (mother and father) and peers (best friend and romantic partner) in adolescence. The current study represents the latest phase of an 18-year longitudinal study, targeting both parents and adolescents.

## Materials and methods

### Participants and procedure

The study sample originally comprised 1095 Finnish families, 972 with singleton pregnancies (513 ART and 473 NC) and 123 families with twins (101 ART and 22 NC) (Vilska *et al.*, 2009). Half (50.7%) of the children were girls. ART families conceived with IVF or ICSI with their own gametes, using fresh or frozen embryo transfer. Reflecting the actual share of public vs. private sector treatments in Finland at that time, about a fourth (26.8%) were recruited from a public infertility clinic in Helsinki University Central Hospital, and the rest were from four private clinics (where treatments also received significant public funding): Family Federation of Helsinki, Turku and Oulu and the Deaconess Institute of Helsinki. This represents about 75% of successful treatments in the participating clinics and about a third of all successful treatments in Finland during the time of the data collection (Poikkeus *et al.*, 2006). The NC group was recruited during a routine ultrasonographic examination from a hospital maternity clinic in Helsinki University Central Hospital. Inclusion criteria for both groups included being Finnish-speaking and, for the control group, being over 25 years old (to match the ages of ART parents who are typically older than average) and having no history of infertility. Even though most participants were from the Finnish capital (Helsinki) region, Finland has national service guidelines, making health care services comparable across the country. More information on the sample and its recruitment can be found in Poikkeus *et al.* (2006), Repokari *et al.* (2005) and Vilska *et al.* (2009).

Mothers and fathers were asked to separately complete questionnaires during the second trimester of pregnancy (T1), child age of 2 months (T2), child age of 12 months (T3) and child age of 7–8 years (T4). At 17–19 years (T5), both adolescents and their parents separately completed electronic questionnaires. For the ART group, research nurses also collected data from the clinics’ patient registries related to their infertility characteristics and other prenatal medical history at T1.

### Measures

*Background variables* consisted of T1 maternal and paternal self-reported education level (unskilled worker =1, skilled worker = 2, low professional = 3, high professional = 4), and ART characteristics (as reported by the research nurse): type of treatment (IVF/fresh, IVF/frozen, ICSI/fresh, ICSI/frozen), etiology of infertility (female, male, combined male and female, or unknown) and duration of infertility (in months). The T2 maternal self-reported (maternal report was used as it was the most comprehensive) child health risk factors: newborn health problems (1 = yes/0 = no), gestational week at birth and birth weight. As newborn health problems (not healthy at birth, gestational week < 37 or birth weight <2500 g) were highly intercorrelated, they were combined into a newborn health risk index (0–3) for further analyses describing the number of risks. At T5, the adolescents self-reported on whether they were currently dating (1 = no, 2 = yes) and their education level. Education level consisted of upper secondary school (non-mandatory school in Finland, a prerequisite for higher education such as university), vocational training, working without a professional training and being unemployed without a professional training. Since 75.9% of adolescents were in upper secondary school, while all the other categories were small (14.2% in vocational training, 3% working and 6.9% being unemployed), the classes were dichotomized into high (i.e. upper secondary school = 1) vs. low education level (the latter consisting of vocational training, working or being unemployed = 2). Adolescent sex (girl = 1, boy = 2) was based on the questionnaire information from mothers and fathers at T1–T4 (all information was combined to minimize missing data). Parental divorce (1 = no/2 = yes) and number of siblings were based on the questionnaire information from mothers, fathers and adolescents at T5 (all information was combined to minimize missing data), and adolescent age at T5 was calculated based on their birthdate (as reported by mothers at T1) and T5 participation date (as recorded electronically when they filled the questionnaire).

*Attachment to mother, father, best friend*  *and romantic partner* was assessed from adolescents with a questionnaire, using the *Experiences in Close Relationships—Relationship Structures* (*ECR-RS*; Fraley *et al.*, 2011) at T5. This is a 9-item questionnaire using a 7-point Likert Scale (Range: 1 = Strongly disagree to 7 = Strongly agree) assessing adolescents’ attachment avoidance (6 items; e.g. ‘I prefer not to show this person how I feel deep down’) and attachment anxiety (3 items; e.g. ‘I’m afraid that this person abandons me’) separately in each of the four relationships. In case the adolescent did not have a current romantic partner, they were asked to report on a former partner, or in case they had never dated, on an imagined partner, according to the standard instructions of the measure (Fraley *et al.*, 2011). Summary variables (Range: 6–42 for avoidance and 3–21 for anxiety) were formed separately for each scale. Scale reliabilities (Cronbach’s alpha) were 0.90 for mother avoidance, 0.89 for mother anxiety, 0.91 for father avoidance, 0.89 for father anxiety, 0.88 for friend avoidance, 0.91 for friend anxiety, 0.87 for partner avoidance and 0.93 for partner anxiety. ECR-RS is derived from ECR-R (Fraley *et al.*, 2000), which uses the same set of questions but measures generalized mental models of attachment (i.e. not directed to specific attachment figures). The reliability and validity of the original ECR-R has been established in several studies for adults (Sibley and Liu, 2004; Sibley *et al.*, 2005) and adolescents (Wilkinson, 2011; Hao *et al.*, 2019) across various cultures. ECR-R is also highly concordant with adult attachment measures using a categorical approach of secure and insecure attachments (Wongpakaran *et al.*, 2021). The validity of ECR-RS in adolescence has been demonstrated by showing meaningful associations with the generalized attachment models measured with ECR-R (Donbaek and Elklit, 2014). Furthermore, in our sample, higher attachment anxiety and attachment avoidance as measured with ECR-RS were related to adolescent mental health and substance use problems (singletons-only study; Flykt *et al.*, 2021). There is no clinical cut-off for the scale, but tentative norms for ECR-R based on a sample of 17 000 adults can be found in: http://labs.psychology.illinois.edu/~rcfraley/measures/ecrr.htm Note: Norms are based on mean variables so to directly compare them with our results using summary variables, anxiety variables need to be divided by 3 (scale 1–7) and avoidance variables need to be divided by 6 (scale 1–7).

### Ethical issues

Ethical permissions were obtained from the ethical board of Helsinki University Central Hospital separately at T1–T3, T4 and T5. At T1–T3, it was also obtained from the participating clinics. Participants gave a written, informed consent separately at each time point, regarding the use of their self-report questionnaires in the research. At T1–T4, the consent was obtained from the parents, and at T5 both parents and the adolescent each gave their own consents regarding their own self-reported questionnaire data. According to Finnish law, parental consent is no longer needed for 17–19-year-old research participants, so parents were only informed about their adolescents’ invitation to participate. At T1, the ART group also gave their informed consent for the collection of their medical registry data from the clinics’ patient registries.

### Analytic strategy

For descriptive analyses, we used non-parametric tests to address the violation of the normality assumption in parametric tests of the attachment variables. Mann–Whitney *U* tests were used to examine the associations of attachment variables with background variables: Adolescent sex, education level, current dating and parental divorce, and Spearman’s correlations to examine their associations with maternal and paternal education level, adolescent age at T5, newborn health risk index (including newborn health, gestational age and birth weight) and number of siblings. In the ART group, we further examined the associations of the treatment type and the etiology of infertility with attachment variables with the Kruskal–Wallis test and the association of duration of infertility and attachment variables with Spearman’s correlations. We then examined the associations between group status (ART twin, ART singleton, NC twin or NC singleton) and background variables using chi square tests and ANOVAs. The background variables significantly associated with attachment variables were used as covariates in the respective models for the research questions.

To answer our research question about differences between ART and NC twins and singletons in attachment variables, multilevel structural equation models (MSEMs) were built with Mplus version 8 (Muthén and Muthén, 1998–2017) using Maximum Likelihood estimation method with robust standard errors (MLR). We used a specific method, partially nested MSEM, which takes into account that twin pairs do not represent independent observations but standard errors for the twins are smaller than standard error for the singletons in our study. Partially nested MSEMs approach allows unbiased results and more reliable comparisons across groups of singletons and twins, by adjusting the standard errors for the twins (Baldwin *et al.*, 2011; Sterba, 2017).

**Figure 1.**
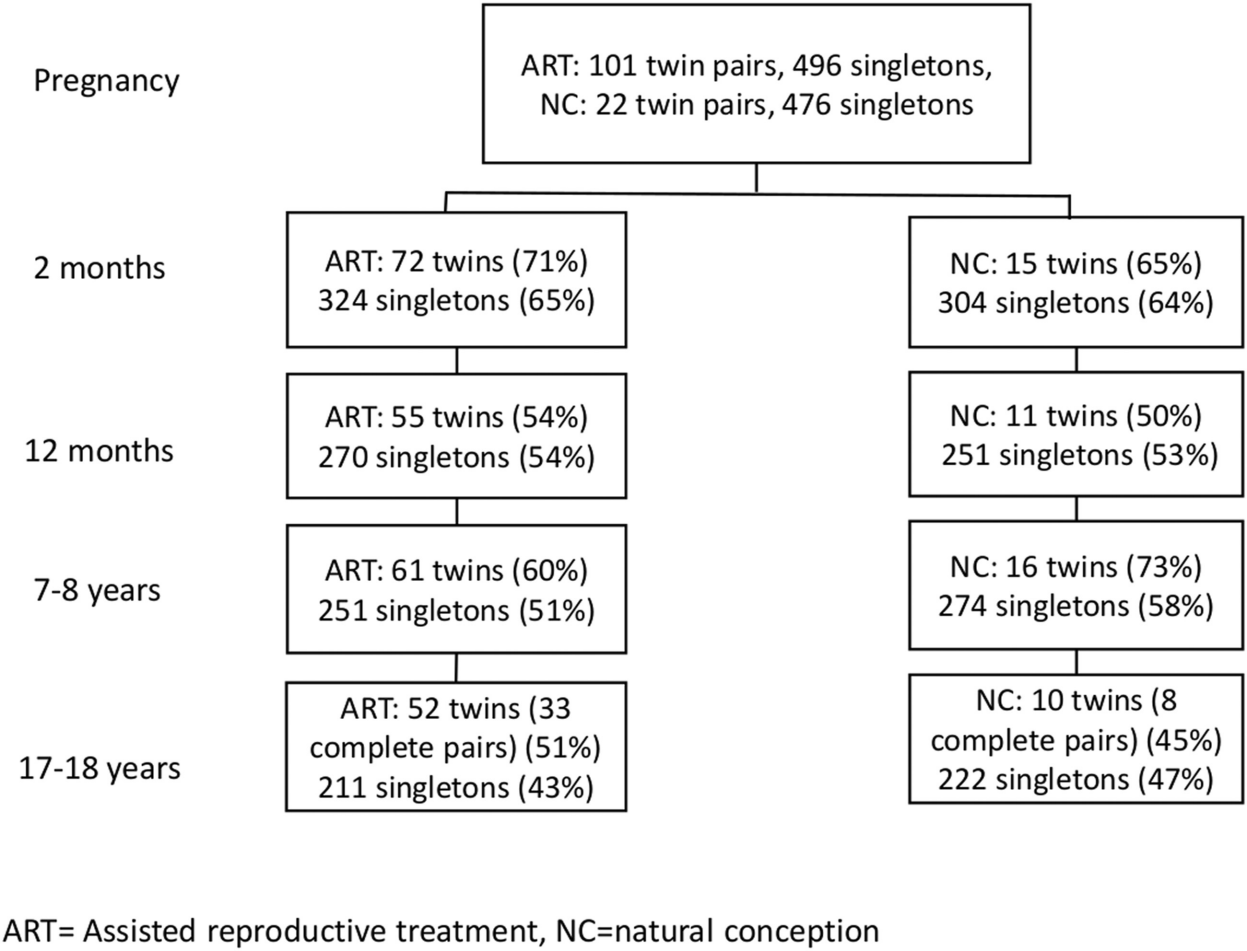
Flow chart showing the participation rates in different phases of the study.

We used multiple imputation to handle missing data, which are appropriate for nested data structures (in our study: data included both twins and singletons), and for use with a combination of continuous and categorical variables. Multiple imputation is an analysis method utilizing all variables in the data to estimate missing values (Dong and Peng, 2013). Multiple imputation was a more reliable method for handling missing values in our data than listwise deletion (i.e. a strategy that would just utilize the existing data), which could lead to severely biased results with high levels of missingness, non-normal distribution of variables and when data are not missing completely at random (Dong and Peng, 2013). Multiple different imputed datasets (10 in our study) were used to form pooled estimates for missing values employing fully conditional specification approach to multiple imputation (Enders, 2017; Enders *et al.*, 2018). Missing data rates for adolescent attachment variables are reported in Supplementary Table I (see also Fig. 1 for the general drop-out rates of the study and in the Results section for the associations of background variables with drop-out at T5).

Adolescent attachment anxiety and avoidance variables (with the exception of attachment avoidance to father, which was normally distributed) were skewed in a way that most values were at the lowest value. To appropriately model this type of data, a censor-inflated regression model was fitted to the data (except for father-avoidance model). Traditional model fit indices cannot be used with partially nested models, so fit indices were not calculated for attachment variables (Muthén and Muthén, 1998–2017). Dummy coded categorical variables were created to allow examination of all pairwise comparisons between the four groups. In the censored regression model, these dummy variables, namely the four groups (ART twins, ART singletons, NC twins and NC singletons), serve as independent variables, and the censored attachment variables (anxiety and avoidance toward mother, father, best friend and romantic partner), serve as the dependent variables.

## Results

### Descriptive statistics

The participation flow chart is presented in Fig. 1. Drop-out at T5 was not associated with group status (ART twin, ART singleton, NC twin, NC singleton), χ^2^(3) = 2.09, *P *=* *0.55, health as newborn, χ^2^(1) = 0.83, *P *=* *0.36, gestational week at birth, t(710) = –0.37, *P *=* *0.71 or child birth weight, t(970) = –1.74, *P *=* *0.082, nor in the ART group, with the etiology (male/female/combined male and female/unknown), χ^2^(3) = 4.80, *P *=* *0.19, or duration of infertility, t(408) = –0.58, *P *=* *0.56, or the type of treatment (IVF/fresh, IVF/frozen, ICSI/fresh, ICSI/frozen), χ^2^(3) = 0.45, *P *=* *0.93. However, higher drop-out was associated with lower T1 maternal and paternal education level, χ^2^(3) = 10.54, *P *=* *0.014 and χ^2^(3) = 13.56, *P* = 0.004 and the child being a boy, χ^2^(1) = 28.67, *P *<* *0.001

Table I shows the means and standard deviations of the attachment summary variables for each group. Table II displays the associations between background variables and the four groups (ART singleton, ART twin, NC singleton and NC twin). ART and NC twins and singletons differed in adolescent age: both NC groups were older than the ART groups, and ART twins were also older than ART singletons. Adolescents in ART singleton families also had fewer siblings than the other three groups. ART and NC twins had more newborn health risks than ART and NC singletons. Within the ART groups, twins were more often conceived with fresh embryo transfer and less often with frozen embryo transfer as compared to singletons.

**Table I:** Means, standard deviations and reliabilities of the attachment variables.

	ART twin		ART singleton		NC twin		NC singleton		Reliability
	M	Sd	M	Sd	M	Sd	M	Sd	Cronbach’s α
Mother avoidance	15.60	8.58	16.14	8.18	16.89	7.20	16.34	8.37	0.90
Mother anxiety	3.80	2.06	4.42	2.93	5.11	4.91	4.15	2.78	0.89
Father avoidance	20.73	9.30	3.81	8.96	23.88	11.34	21.89	9.30	0.91
Father anxiety	5.61	4.37	5.09	19.81	6.50	4.47	4.79	3.21	0.89
Best friend avoidance	14.37	7.92	13.46	6.94	14.06	6.53	12.67	6.91	0.88
Best friend anxiety	6.40	4.61	6.69	4.45	6.76	5.08	6.29	4.21	0.91
Partner avoidance	11.22	6.18	10.80	5.66	12.65	7.67	11.55	6.41	0.87
Partner anxiety	5.12	3.24	6.21	4.51	4.59	3.45	6.44	4.70	0.93

NC, natural conception.

Summary variables range from 3 to 21 for attachment anxiety variables and from 6 to 42 for attachment avoidance variables.

**Table II.** Associations between group status (ART twin, ART singleton, NC twin, NC singleton) and background variables.

	ART twin		ART singleton		NC twin		NC singleton			
	n	%	n	%	n	%	n	%	χ^2^(df)	*P*
Adolescent sex									0.83 (3)	0.84
Girl	77	53.5	189	51.2	18	51.4	212	49.3		
Boy	67	46.5	180	48.8	17	48.6	218	50.7		
Education level									0.70 (3)	0.87
High	67	78.8	161	76.3	13	72.2	166	74.8		
Low	18	21.2	50	23.7	5	27.8	56	25.2		
Parental divorce									4.83 (3)	0.19
No	72	73.5	197	72.7	8	50	182	68.4		
Yes	26	26.5	74	27.3	8	50	84	31.6		
Current dating									3.08 (3)	0.38
No	65	78.3	146	69.2	14	77.8	154	69.7		
Yes	18	21.7	65	30.8	4	22.2	67	30.3		

	ART twin		ART singleton		NC twin		NC singleton			
	n	%	n	%	n	%	n	%	χ^2^(df)	*P*
Mother’s education									9.25 (3)	0.42
Master’s degree	21	25.9	105	27.5	6	35.3	126	33.2		
Bachelor	31	38.3	160	41.9	6	35.3	165	43.4		
Vocational school	18	22.2	68	17.8	3	17.6	50	13.2		
No training	11	13.6	49	12.8	2	11.8	39	10.3		
Father’s education									12.14 (3)	0.21
Master’s degree	21	26.9	117	31.9	4	30.7	129	36.2		
Bachelor	17	21.8	107	29.2	3	23.1	108	30.3		
Vocational school	27	34.6	105	28.6	4	30.8	77	21.6		
No training	13	16.7	38	10.4	2	15.4	42	11.8		
Treatment type^1^									9.83 (3)	**0.02**
IVF fresh	44	53	152	40.4						
ICSI fresh	21	25.3	74	19.7						
IVF frozen	13	15.7	101	26.9						
ICSI frozen	5	6	49	13						
Etiology of infertility^1^									1.36 (3)	0.71
Female	23	33.8	106	32.4						
Male	18	26.5	74	22.6						
Combined	17	25	80	24.5						
Unknown	10	14.7	67	20.5						

	ART twin		ART singleton		NC twin		NC singleton			
	M	Sd	M	Sd	M	Sd	M	Sd	F (df)	*P*
Newborn risk index	1.21^a^	0.07	0.18^b^	0.03	1.27^a^	0.15	0.11^b^	0.03	92.29 (3706)	**<0.001**
Adolescent T5 age	17.69^a^	0.05	17.56^b^	0.03	17.94^c^	0.10	17.89^c^	0.03	23.74 (3532)	**<0.001**
Number of siblings	1.53^a^	0.11	1.27^b^	0.07	1.90^a^	0.24	1.67^a^	0.07	7.07 (3 637)	**<0.001**
Duration of infertility^1^	54.58	3.83	56.26	1.78					0.16 (1408)	0.69

NC, natural conception; df, degrees of freedom.

^1^ Analyses performed only in the ART groups. Statistically significant *P*-values (*P* < 0.05) are in bold type.

^a,b^ Groups differ significantly from each other (*P*<0.05). χ^2^ refers to chi square test. F refers to Univariate Anovas. Differences in n’s are due to missing values.

Table III shows the associations between background variables and adolescent attachment variables. Adolescent sex was significantly associated with attachment avoidance toward mother, best friend and partner, with boys showing higher avoidance than girls. Concerning adolescent education level, those with higher education level (i.e. enrolled in non-mandatory upper secondary school in Finland) showed lower attachment anxiety toward their mother, father and romantic partner and lower attachment avoidance toward their best friend and partner than those with lower education level (i.e. who were in vocational training, working or unemployed). Parental divorce was significantly associated with higher attachment avoidance and attachment anxiety toward father. Current dating was associated with lower attachment avoidance toward mother, best friend and romantic partner. Older adolescent age was associated with higher attachment avoidance and attachment anxiety toward father. Furthermore, higher newborn risk index scores were associated with higher attachment anxiety toward mother and father. The background variables significantly associated with attachment variables were used as covariates in the respective models. The concordance between twin pairs in attachment variables was examined with intra-class correlations (Table IV), showing significant concordances in attachment anxiety and avoidance toward mother and attachment avoidance toward father as well as attachment anxiety toward best friend.

**Table III:** The associations between background variables and adolescent attachment variables.

	Mother avoidance				Mother anxiety			Father avoidance			Father anxiety		
	Md	U	*P*		Md	U	*P*	Md	U	*P*	Md	U	*P*
Sex		40 237	**0.002**			33 969	0.57		31 048	0.18		31 477	0.21
*Girl* (n = 313)	13.0				3.0			21.0			3.0		
*Boy* (n = 222)	16.0				3.0			20.0			3.0		
Education		25 773	0.86			28 606	**0.028**		26 453	0.31		28 444	**0.006**
*High* (n = 404)	14.0				3.0			20.0			3.0		
*Low* (n = 120)	14.0				3.0			21.0			3.0		
Divorce		25 177	0.63			26 030	0.15		27 841	**0.004**		27 271	**0.004**
*No* (n = 366)	14.0				3.0			19.0			3.0		
*Yes* (n = 128)	15.0				3.0			22.0			3.0		
Current dating		24 542	**0.004**			29 329	0.86		27 868	0.86		28 287	0.92
*No* (n = 368)	15.0				3.0			20.0			3.0		
*Yes* (n = 153)	13.0				3.0			20.0			3.0		

	χ^2^(3)		*P*		χ^2^(3)		*P*	χ^2^(3)		*P*	χ^2^(3)		*P*
Treatment type^1^	4.12		0.25		1.51	0.68	2.02		0.57	2.45		0.48
Etiology of infertility^2^	2.40		0.49	5.42		0.14	1.42		0.70	1.87		0.60

	Friend avoidance				Friend anxiety			Partner avoidance			Partner anxiety		
	Md	U		*P*	Md	U	*P*	Md	U	*P*	Md	U	*P*
Sex		45 440		**<0.001**		32 899	0.40		38 790	**0.001**		34 561	0.38
*Girl* (n = 313)	10.0				5.0			8.0			3.0		
*Boy* (n = 222)	14.5				5.0			11.0			4.0		
Education		30 524		**0.001**		25 486	0.87		28 907	**0.001**		27 507	**0.017**
*High* (n = 404)	11.0				5.0		9.0				3.0		
*Low* (n = 120)	14.0				5.0		11.0				6.0		
Divorce		24 367		0.82		25 347	0.35		24 588	0.39		25 441	0.12
*No* (n = 366)	11.0				5.0			9.0			3.0		
*Yes* (n = 128)	12.0				5.0			10.0			4.5		
Current dating		23 771		**0.001**		27 179	0.27		22 718	**<0.001**		28 708	0.71
*No* (n = 368)	12.0				5.0			10.0			3.0		
*Yes* (n = 153)	9.5				5.0			8.0			4.0		

	χ^2^(3)		*P*		χ^2^(3)		*P*	χ^2^(3)		*P*	χ^2^(3)		*P*
Treatment type^1^	3.46			0.33	1.40		0.71	2.57		0.46	1.10		0.78
Etiology of infertility^2^	1.67			0.65	0.31		0.96	1.56		0.67	0.69		0.88

	Mother anxiety		Mother avoidance		Father anxiety		Father avoidance		Friend anxiety		Friend avoidance		Partner anxiety		Partner avoidance	
	R	*P*	R	*P*	R	*P*	R	*P*	R	*P*	R	*P*	R	*P*	R	*P*
Mother’s education	0.07	0.17	0.002	0.97	0.07	0.18	–0.003	0.96	0.005	0.92	–0.008	0.88	0.01	0.78	–0.05	0.32
Father’s education	0.07	0.14	–0.03	0.57	0.08	0.10	0.04	0.42	0.04	0.42	0.02	0.64	0.03	0.57	–0.02	0.76
Newborn risk index	0.10	**0.05**	–0.01	0.83	0.21	**<0.001**	0.10	0.055	0.03	0.53	0.008	0.87	0.05	0.36	0.04	0.43
Adolescent T5 age	0.008	0.86	0.03	0.48	0.10	**0.025**	0.09	**0.04**	0.009	0.84	0.01	0.79	0.05	0.23	0.01	0.81
Number of siblings	0.01	0.77	–0.003	0.94	0.03	0.51	–0.001	0.98	–0.01	0.82	–0.003	0.94	–0.014	0.76	0.07	0.12
Duration of infertility^a^	0.007	0.92	–0.01	0.84	–0.02	0.82	–0.06	0.43	0.03	0.70	0.11	0.13	–0.02	0.83	0.03	0.64

^1^ Measured only in the ART groups. Treatment types are IVF fresh, ICSI fresh, IVF frozen and ICSI frozen.

^2^ Measured only in the ART groups. Etiologies of infertility include female, unknown, male, and combined male and female. Md, Median. U’s refer to Mann–Whitney U tests. χ^2^ refers to Kruskal–Wallis test. R’s refer to Spearman’s correlation coefficients. Statistically significant *P*-values (*P* < 0.05) are in bold type.

**Table IV:** Intra-class correlations within twin pairs.

	ICC
Adolescent attachment avoidance to mother	0.72***
Adolescent attachment anxiety to mother	0.70***
Adolescent attachment avoidance to father	0.69***
Adolescent attachment anxiety to father	0.29
Adolescent attachment avoidance to best friend	0.41
Adolescent attachment anxiety to best friend	0.43*
Adolescent attachment avoidance to partner	–0.001
Adolescent attachment anxiety to partner	0.30

ICC, intra-class correlations.

* *P* < 0.05,

*** *P* < 0.001. Statistically significant *P*-values (*P* < 0.05) are in bold type.

### Adolescent attachment toward parents and peers in ART and NC singleton and twins

The results in Table V indicate that there were no differences between ART twins, ART singletons, NC twins and NC singletons in attachment avoidance and attachment anxiety toward the mother. However, NC singletons showed more attachment anxiety toward their father than ART twins and marginally more than NC twins. Furthermore, ART singletons showed more attachment avoidance toward their father than NC singletons, and marginally more than NC twins.

**Table V:** Adolescent attachment in ART and NC twins and singletons.

	Adolescent’s attachment anxiety to mother^1^					Adolescent’s attachment avoidance to mother^2^				
*Contrasts*	B	β	SE (B)	CI (B)	*P*	B	β	SE(B)	CI (B)	*P*
ART twin vs. ART singleton	0.53	0.23	0.35	[–0.16, 1.21]	0.13	1.08	0.14	1.33	[–1.53, 3.68]	0.42
ART twin vs. NC twin	0.81	0.10	0.92	[–1.00, 2.61]	0.38	1.08	0.04	2.83	[–4.45, 6.62]	0.70
ART twin vs. NC singleton	0.17	0.08	0.42	[–0.65, 0.99]	0.72	1.21	0.15	1.12	[–1.69, 3.41]	0.28
ART singleton vs. NC twin	0.27	0.04	0.85	[–1.39, 1.94]	0.75	0.06	0.002	2.48	[–4.81, 4.92]	0.98
ART singleton vs. NC singleton	–0.36	–0.16	0.34	[–1.03, 0.31]	0.29	0.17	0.02	0.98	[–1.75, 2.09]	0.86
NC twin vs. NC singleton	–0.67	–0.30	1.03	[–2.69, 1.34]	0.52	0.58	0.08	2.83	[–4.96, 6.12]	0.84
	Between-subject R ^2^s range between 0.07 and 0.08					Between subject-R^2^s = 0.09				

	Adolescent’s attachment anxiety to father^3^					Adolescent’s attachment avoidance to father^4^				
	B	β	SE(B)	CI (B)	*P*	B	β	SE(B)	CI (B)	*P*
ART twin vs. ART singleton	–0.77	–0.32	0.50	[–1.75, 0.22]	0.13	–1.54	–0.20	1.27	[–4.04, 0.95]	0.23
ART twin vs. NC twin	0.69	0.09	0.96	[–1.18, 2.56]	0.47	3.40	0.12	2.47	[–1.44, 8.24]	0.17
ART twin vs. NC singleton	–1.24	–0.53	0.43	[–2.09, –0.04]	**0.004**	0.60	0.06	1.10	[–1.55, 2.75]	0.58
ART singleton vs. NC twin	1.46	0.20	1.10	[–0.69, 3.61]	0.18	4.96	0.17	2.59	[–0.12, 10.04]	0.055^+^
ART singleton vs. NC singleton	–0.48	–0.22	0.39	[–1.23, 0.28]	0.22	2.16	0.26	0.79	[0.61, 3.72]	**0.006**
NC twin vs. NC singleton	–1.89	–0.90	1.008	[–3.87, 0.09]	0.06^+^	–1.76	–0.24	2.86	[–7.36, 3.84]	0.54
	Between-subject R^2^s range between 0.54 and 0.61					Between-subject R^2^s = 0.24				

	Adolescent’s attachment anxiety to father^5^					Adolescent’s attachment avoidance to father^6^				
	B	β	SE(B)	CI (B)	*P*	B	β	SE(B)	CI (B)	*P*
ART twin vs. ART singleton	0.27	0.09	0.77	[–1.25, 1.78]	0.73	–0.67	–0.15	0.87	[–2.37, 1.03]	0.44
ART twin vs. NC twin	0.37	0.04	1.009	[–1.61, 2.35]	0.71	–0.36	–0.02	1.58	[3.46, 2.74]	0.82
ART twin vs. NC singleton	–0.15	–0.08	0.62	[–1.36, 1.07]	0.81	–1.73	–0.39	0.95	[–3.60, 0.14]	0.07^+^
ART singleton vs. NC twin	0.07	0.02	0.90	[–1.68, 1.83]	0.94	0.27	0.02	1.68	[–3.02, 3.55]	0.88
ART singleton vs. NC singleton	–0.44	–0.16	0.45	[–1.33, 0.45]	0.33	–1.11	–0.26	0.80	[–2.68, 0.46]	0.17
NC twin vs. NC singleton	–0.87	–0.34	1.00	[–2.83, 1.09]	0.38	–1.81	–0.43	1.65	[–5.05, 1.43]	0.27
	Between-subject R^2^s range between 0.06 and 0.07					Between-subject R^2^s range between 0.27 and 0.28				

	Adolescent’s attachment anxiety to father^7^					Adolescent’s attachment avoidance to father^8^				
	B	β	SE(B)	CI (B)	*P*	B	β	SE(B)	CI (B)	*P*
ART twin vs. ART singleton	0.73	0.33	0.49	[–0.23, 1.69]	0.13	–0.42	–0.10	0.93	[–2.25, 1.41]	0.65
ART twin vs. NC twin	–0.98	–0.11	1.34	[–3.61, 1.65]	0.47	0.59	0.08	1.73	[–2.79, 3.98]	0.73
ART twin vs. NC singleton	1.17	0.52	0.88	[–0.56, 2.89]	0.19	–1.04	0.18	1.56	[–1.05, 1.99]	0.55
ART singleton vs. NC twin	–1.70	–0.20	1.21	[–4.06, 0.67]	0.16	0.98	0.12	1.49	[–1.95, 3.90]	0.51
ART singleton vs. NC singleton	0.44	0.18	0.39	[–0.32, 1.20]	0.25	0.83	0.26	0.57	[–0.28, 1.95]	0.14
NC twin vs. NC singleton	1.81	0.83	1.06	[–0.27, 3.90]	0.09^+^	–0.48	–0.17	6.42	[–13.06, 12.10]	0.94
	Between-subject R^2^s range between 0.35 and 0.42					Between-subject R^2^s range between 0.63 and 0.64				

NC, natural conception. Statistically significant *P*-values (*P* < 0.05) are in bold type. ^+^*P* < 0.10. The same covariates were used both within- and between level. Higher values represent higher attachment anxiety/avoidance.

^1^ Covaried with adolescent education level and newborn health risk index.

^2^ Covaried with adolescent sex and current dating.

^3^ Covaried with adolescent education level, parental divorce, adolescent age, and newborn health risk index.

^4^ Covaried with divorce and adolescent age.

^5^ No covariates.

^6^ Covaried with adolescent sex, education level and current dating.

^7^ Covaried with adolescent education level.

^8^ Covaried with adolescent sex, education level and current dating.

Concerning attachment in peer relationships (best friend and romantic partner), no statistically significant effects were found between any of the four groups. However, two marginally significant trends emerged, suggesting that NC singletons showed more attachment avoidance toward best friend than ART twins, and NC twins showed more attachment anxiety toward romantic partners than NC singletons. None of the covariates were significant for any of the parental or peer models (see Supplementary Table II). To view the results without covariates, please see Supplementary Table III.

## Discussion

This study examined whether ART and NC singletons and twins differed from each other in adolescent attachment toward parents (mother and father) and peers (best friend and romantic partner). Interestingly, adolescent backgrounds of ART and twin status showed distinct effects on adolescent attachment anxiety and attachment avoidance toward the father, but not with attachment to mother or with peers. Twins in general, and most clearly ART twins, showed lower attachment anxiety toward father compared to NC singletons. Conversely, a background of ART was associated with higher attachment avoidance toward the father compared to NC adolescents (both singletons and twins), but this only applied to ART singletons.

Our findings concur with earlier studies showing that ART singletons experience at least as warm and harmonious relationships to their mothers in adolescence as NC singletons (Colpin and Bossaert, 2008; Goisis and Palma, 2021). However, our findings extend this prior work to twins, namely, by showing no differences in attachment anxiety or avoidance toward the mother between ART and NC singletons and twins. It appears that despite the stressful nature of infertility treatments, or the stress of parenting twins, families conceiving with ART are resilient, and their twin and singleton children are as securely attached to their mothers in late adolescence as NC children. Our results also indicated generally very low levels of attachment anxiety and attachment avoidance toward the mother being typical for all adolescents, as indicated by the skewness of distribution of attachment variables.

Our findings on adolescents’ attachment to their father were especially interesting, as ART and twin status showed different dynamics related to attachment anxiety and attachment avoidance. Compared to NC singletons, both ART and NC twin groups displayed lower attachment anxiety, that is, less over-dependent relationships with their fathers, where there are worries of paternal availability. This may be because in the challenging twin context, parents are less likely to overprotect, and have a greater tendency to foster autonomy in their twin children. They simply have to because their energies are distributed across multiple children. While it is not absolutely clear why this is the case for fathers but not the mothers, we believe that the fathers feel this ‘limited resource’ issue of time and energy even more than the mothers. There may also be something protective in the twin relationship itself: for example, at least adult twins report especially close attachments with each other (Tancredy and Fraley, 2006; Fraley and Tancredy, 2012), which could make them less prone to attachment anxiety or over-dependence toward other family members. While this is a plausible explanation for why twins do not show attachment anxiety (or over-dependence) with their parents, future studies might also examine the role of twins’ attachment toward each other to more comprehensively understand whole-family dynamics in twin families.

In the singleton context, ART singletons did not differ from any of the other groups, suggesting that ART singletons do not display higher attachment anxiety toward their father. These findings are important, given that some early childhood studies have reported early family-level enmeshment in ART singletons (Cairo *et al.*, 2012; Lindblom *et al.*, 2014) that could be associated with attachment anxiety, which is also characterized by too much dependence on others at the expense of one’s autonomy. Thus, in adolescence, such over-dependence or enmeshment does not appear to be the case for ART singletons, potentially indicating this is an issue for ART families only in the earliest years, if at all. However, our findings on attachment avoidance to father were the opposite and will be discussed next.

Attachment avoidance typically indicates difficulties in relying on others or have others rely on them, and not easily expressing one’s needs and vulnerabilities (Fraley *et al.*, 2011). Some studies show that ART mothers form an especially close relationship with their children (Goisis and Palma, 2021). It is possible that this may decrease the father’s participation in child care, potentially leading to more distant father–child relationships. It is also clinically interesting that it is specifically ART fathers, but not mothers, who may show long-term parenting vulnerability after infertility, perhaps indicating that fathers receive especially inadequate support. Recent qualitative studies indicate that at least during infertility treatments, men experience less agency, involvement and support than women, and are culturally expected to be the silent strong ones who support their spouse (Hanna and Gough, 2017; Sylvest *et al.*, 2018). Interestingly, previous findings in our earlier study stemming from the child’s first year similarly indicated that especially the ART fathers were vulnerable for disappointments in their parent–child relationship intimacy when facing additional distress (Flykt *et al.*, 2014). Although being conceived through ART seemed to increase adolescent attachment avoidance toward father, this was only true among singletons, and again, this may be because the twin context may create some sort of buffer for parent–child relationships.

Our findings differ from Anderson *et al.* (2017), who found that 12-year-old ART twins showed more problematic observed interactions with their fathers than ART singletons, and from the self-report studies of Colpin and Bossaert (2008) and Goisis and Palma (2021), who found no differences between ART and NC singletons. There may be several reasons for the different findings. First, no previous study has included all four groups of ART and NC twins and singletons in the same study. Second, our study is the first to specifically examine adolescent attachment, which is conceptually close to, but not identical to measures of the parent–adolescent relationship. For example, Goisis and Palma (2021) measured self-reported warmth and conflict as indices of relationship quality. However, in the realms of attachment, anxious attachment can be warm, but over-dependent, or dyads with avoidant attachment may be low in reported conflict, as avoidantly attached individuals typically suppress or downplay negative emotions. Third, it is possible that self-reports in general are less reliable in assessing family relationships. Anderson *et al.'s* study is, to our knowledge, the only study using an objective, observational design of the parent–child relationship. In attachment research, the golden standard measure for objective assessment would be using Adult Attachment Interview (AAI; Main *et al.*, 2002). Our self-report-based attachment results should thus ideally be verified with AAI, although it is very time-consuming in larger samples.

Related to adolescent attachment to peers (best friend and romantic partner), no significant differences emerged. Overall, the attachment relationships to best friend and romantic partner showed in average low attachment avoidance and attachment anxiety, further indicating that neither individuals conceived with ART nor twins are likely to show problems in peer attachment. Since this is, to our knowledge, the first study on peer attachment after ART, more research is warranted. However, our results support the findings of Golombok *et al.* (2009) in that conceiving with ART is not associated with risks for adolescent peer relationships.

Some of our descriptive statistics were also interesting. Especially, there was an association between newborn health risks (prematurity, birth weight and newborn health problems) and higher adolescent attachment anxiety toward mother and father. A previous study by Hallin and Stjernqvist (2011) similarly found that premature infants showed less secure and especially anxious attachment to their parents in adolescence. Since newborn health risks are much more common in twins and to some extent also among ART singletons, these should be taken into account in future attachment studies. It is possible that these characteristics could mediate findings of ART or twinhood on relationship problems with parents, especially regarding anxious attachment which may be related to parental over-indulgement or other over-protection sometimes described in ART parents (Owen and Golombok, 2009). Furthermore, parental divorce formed a general risk for less secure attachment to father, and current dating indicated generally more secure attachments, suggesting that their role as mediators or moderators could be examined in future studies.

Our prospective, 18-year longitudinal study was, to our knowledge, the first to compare the four groups of ART and NC twins and singletons on the outcomes of relationship to parents and peers. Additionally, it is the first study to assess attachment beyond infancy as an outcome of ART. Our study was also unique in taking into account the statistical dependence of twins, and making appropriate statistical corrections, which is rarely done in twin studies.

The main limitation of the study was the relatively small sample size in twin groups, especially NC twins, which was exacerbated by drop-out over the 18 years of the longitudinal study. There was also more drop-out among boys and families with lower parental education level. However, highly sophisticated methods were used to replace missing data, and based on earlier simulation studies on multi-level partially clustered data (Baldwin *et al.*, 2011; Arend and Schäfer, 2019), the power in the current analyses was deemed adequate to detect small to medium sized effects between groups. The results should still ideally be replicated with larger twin group sizes, which would also enable analyses of the possible mediating factors. In our sample, ART singletons were younger and had fewer siblings than ART twins and NC adolescent, and twins overall had more newborn health risks than singletons. Ideally, the role of these variables as mediators should be examined, along with factors such as earlier parent–child relationship quality or other family psychosocial factors. Future studies could also measure attachment in ART and NC twins and singletons in multiple time points.

A second limitation concerns the generalizability of the results. All participants were native Finnish-speakers, and in Finland, the expenses of infertility treatments are also to a large extent publicly funded, making the treatments available for families with lower socio-economic status (SES) than in many other countries. However, there is evidence that at least early treatment-related outcomes are not associated with family SES (Räisänen *et al.*, 2013), suggesting that our findings may still be relevant for countries where treatments are not publicly funded. Third, although ART-related factors were derived from patient registries of the clinics, we used maternal report regarding newborn health, as we did not have access to patient registry data, which could be more reliable. Finally, some caution may need to be used in interpreting the results regarding the romantic attachment, as only 28.9% of the adolescents were currently in a romantic relationship. Asking about romantic relationship even among those not currently dating is part of the standard instructions of the measure (Fraley *et al.*, 2011), based on the theoretical idea of attachment as generalized mental models not based on the current relationship only but with roots in earlier close relationships, including those with one’s parents and earlier partners (Fraley and Shaver, 2000).

Previous studies (e.g. Schwarz *et al.*, 2015) have also suggested differences in close relationships between monozygotic and dizygotic twins, and this should be examined further in terms of attachment, as information on this was lacking in our study. It is also possible that factors such as adolescent’s knowledge of their ART origins affects their relationship with parents, which could be an important topic of future studies. Finally, our results also raise the question of father–child relationships after ART as potentially having more long-term vulnerability, suggesting that father’s experiences and fathering after ART should be clinically recognized along with maternal experiences. Although male-factor infertility is generally considered a psychological risk for fathers (Wischmann and Thorn, 2013), it was not associated with adolescent attachment in our study, suggesting that infertility in general may have some long-standing consequences for fathers who do not receive enough support.

Overall, the results contribute importantly to the accumulating long-term evidence that neither ART treatments nor being a twin place adolescent psychosocial development at a significant risk. Infertility and its treatments, as well as parenting young twins who often have medical problems, are typically stressful experiences. It would be a relief for parents to be informed about the safety of the treatments also in terms of the long-term psychosocial development of the children. However, our findings suggest that fathers conceiving with ART need to be kept in mind by health care providers to ensure they receive the same support in the pre- and post-natal services as the mothers. Although infertility-related counseling services for men have improved after our data collection (20 years ago), these services are still more directed to women (Petok, 2015). Especially, early support in infertility clinics seems vital, as men are less likely to seek mental health services or to rely on their friends and family as a source of support (Fisher and Hammarberg, 2012). Yet, it should be emphasized that the differences found in attachments to father were not very comprehensive: ART singletons only differed from NC adolescents in higher attachment avoidance to father. Furthermore, while twins often experience early medical risks such as prematurity, they were not at higher risk for attachment insecurity in our sample, contributing to the evidence that ART does not increase the risks for twins in the psychosocial realm.

## Supplementary data

Supplementary data are available at *Human Reproduction Open* online.

## Data availability

The data underlying this article cannot be shared publicly due to the privacy of individuals who participated in the study. The data will be shared on reasonable request to the corresponding author.

## Authors’ roles

Participation in study design: A.T., R.-L.P., M.S.F., M.V., J.L., M.P. and P.P. Execution: R.-L.P., M.S.F., M.V., J.L. Analysis: M.P., J.M., M.S.F. Interpretation, manuscript drafting and critical discussion: M.S.F., M.P., M.V., J.L., J.M., A.T., P.P., Z.B., R.-L.P.

## Funding

Funding for the study was obtained from the Academy of Finland (grant number 2501308988), the Juho Vainio Foundation for the Miracles of Development research project (https://projects.tuni.fi/kehi/project/) and the Finnish Cultural Foundation to Marjo Flykt.

## Conflict of interest

The authors declare no conflict of interest.

## Supplementary Material

hoac012_Supplementary_Data
