# Supplementary material for: Adolescent attachment to parents and peers in singletons and twins born with assisted and natural conception
Source: Hum Reprod Open. 2022 Mar 8;2022(2):hoac012. doi: 10.1093/hropen/hoac012 (PMC8994490; doi:10.1093/hropen/hoac012)
Supplement: hoac012_Supplementary_Data [file hoac012_supplementary_data.docx]

Supplementary Table I: *Percentages and numbers of missing data in each group for T5 adolescent attachment variables*

|  | ART twin | ART singleton | NC twin | NC singleton |
| --- | --- | --- | --- | --- |
| Mother avoidance | 57.9% (117) | 57.5% (286) | 59.1% (26) | 53.6% (255) |
| Mother anxiety | 57.9% (117) | 57.5% (286) | 59.1% (26) | 53.6% (255) |
| Father avoidance | 59.4% (120) | 58.1% (289) | 63.6% (28) | 54.2% (258) |
| Father anxiety | 59.4% (120) | 58.1% (289) | 63.6% (28) | 54.2% (258) |
| Best friend avoidance | 58.9% (119) | 57.5% (286) | 61.4% (27) | 53.6% (255) |
| Best friend anxiety | 58.9% (119) | 57.5% (286) | 61.4% (27) | 53.6% (255) |
| Partner avoidance | 59.4%(120) | 58.4% (290) | 61.4% (27) | 54.2% (258) |
| Partner anxiety | 59.4%(120) | 58.4% (290) | 61.4% (27) | 54.2% (258) |

*Note.* ART = assisted reproductive treatment. NC = natural conception.

| Supplementary Table II: *Covariate results from the main analyses* | | | | | |
| --- | --- | --- | --- | --- | --- |
|  | **Adolescent’s attachment anxiety to mother** | | | | |
| *Contrasts* | B | β | SE(B) | CI (B) | p |
| **ART twin as reference** |  |  |  |  |  |
| *Within-level covariates* |  |  |  |  |  |
| Education | 0.72 | 0.28 | 0.91 | [-1.06, 2.51] | 0.43 |
| Newborn health risk | -0.03 | -0.04 | 0.13 | [-0.28, 0.22] | 0.82 |
| *Between level-covariates* |  |  |  |  |  |
| Education | -0.12 | -0.09 | 0.95 | [-1.98, 1.75] | 0.90 |
| Newborn health risk | -0.02 | -0.04 | 0.13 | [-0.27, 0.24] | 0.91 |
| **ART singleton as reference** |  |  |  |  |  |
| *Within-level covariates* |  |  |  |  |  |
| Education | 0.65 | 0.25 | 1.06 | [-1.43, 2.72] | 0.54 |
| Newborn health risk | -0.05 | -0.06 | 0.14 | [-0.31, 0.22] | 0.74 |
| *Between level-covariates* |  |  |  |  |  |
| Education | -0.03 | -0.02 | 1.11 | [-2.20, 2.14] | 0.98 |
| Newborn health risk | 0.001 | 0.004 | 0.14 | [-0.28, 0.28] | 1.00 |
| **NC twin as reference** |  |  |  |  |  |
| *Within-level covariates* |  |  |  |  |  |
| Education | 0.63 | 0.24 | 1.40 | [-2.12, 3.39] | 0.65 |
| Newborn health risk | -0.05 | -0.07 | 0.18 | [-0.40, 0.30] | 0.80 |
| *Between level-covariates* |  |  |  |  |  |
| Education | -0.02 | -0.01 | 1.47 | [-2.91, 2.87] | 0.99 |
| Newborn health risk | 0.002 | 0.003 | 0.19 | [-.38, .38] | 0.99 |

|  | **Adolescent’s attachment avoidance to mother** | | | | | |
| --- | --- | --- | --- | --- | --- | --- |
|  | B | β | SE(B) | CI (B) | | p |
| **ART twin as reference** |  |  |  |  | |  |
| *Within-level covariates* |  |  |  |  | |  |
| Child sex | 0.97 | 0.14 | 10.53 | [-19.66, 21.61] | | 0.93 |
| Current dating | -1.61 | -0.23 | 10.92 | [-23.01, 19.79] | | 0.88 |
| *Between level-covariates* |  |  |  |  | |  |
| Child sex | 0.69 | 0.17 | 11.73 | [-22.31, 23.68] | | 0.95 |
| Current dating | -0.89 | -0.22 | 11.84 | [-24.11, 22.32] | | 0.94 |
| **ART singleton as reference** |  |  |  |  | |  |
| *Within-level covariates* |  |  |  |  | |  |
| Child sex | 0.95 | 0.13 | 7.31 | [-13.37, 15.27] | | 0.90 |
| Current dating | -1.64 | -0.23 | 7.59 | [-16.52, 13.24] | | 0.83 |
| *Between level-covariates* |  |  |  |  | |  |
| Child sex | 0.72 | 0.18 | 8.13 | [-15.22, 16.66] | | 0.93 |
| Current dating | -0.86 | -0.22 | 8.26 | [-17.04, 15.33] | | 0.92 |
| **NC twin as reference** |  |  |  |  | |  |
| *Within-level covariates* |  |  |  |  | |  |
| Child sex | 0.97 | 0.13 | 2.05 | [-3.04, 4.97] | | 0.64 |
| Current dating | -1.62 | -0.23 | 2.84 | [-7.18, 3.95] | | 0.57 |
| *Between level-covariates* |  |  |  |  | |  |
| Child sex | 0.70 | 0.18 | 2.24 | [-3.69, 5.08] | | 0.76 |
| Current dating | -0.89 | -0.22 | 3.05 | [-6.86, 5.08] | | 0.77 |
|  | **Adolescent’s attachment anxiety to father** | | | | | |
|  | B | β | SE(B) | CI (B) | | p |
| **ART twin as reference** |  |  |  |  | |  |
| *Within-level covariates* |  |  |  |  | |  |
| Education | 0.99 | 0.27 | 1.50 | [-1.95, 3.94] | | 0.51 |
| Divorce | -1.30 | -0.36 | 0.84 | [-2.95, .35] | | 0.12 |
| Age | -0.68 | -0.09 | 1.55 | [-3.71, 2.35] | | 0.66 |
| Newborn health risk | -0.05 | -0.05 | 0.15 | [-0.34, 0.24] | | 0.74 |
| *Between level-covariates* |  |  |  |  | |  |
| Education | 0.56 | 0.40 | 1.42 | [-2.22, 3.35] | | 0.69 |
| Divorce | 0.48 | 0.40 | 0.91 | [-1.31, 2.27] | | 0.60 |
| Age | 0.75 | 0.19 | 1.69 | [-2.55, 4.05] | | 0.66 |
| Newborn health risk | 0.05 | 0.14 | 0.12 | [-0.19, 0.30] | | 0.67 |
| **ART singleton as reference** |  |  |  |  | |  |
| *Within-level covariates* |  |  |  |  | |  |
| Education | 0.96 | 0.26 | 3.09 | [-5.09, 7.01] | | 0.76 |
| Divorce | -1.30 | -0.36 | 1.84 | [-4.91, 2.31] | | 0.48 |
| Age | -0.67 | -0.09 | 2.05 | [-4.68, 3.35] | | 0.75 |
| Newborn health risk | -0.05 | -0.05 | 0.16 | [-0.36, 0.26] | | 0.74 |
| *Between level-covariates* |  |  |  |  | |  |
| Education | 0.59 | 0.44 | 3.18 | [-5.64, 6.83] | | 0.85 |
| Divorce | 0.48 | 0.43 | 2.00 | [-3.45, 4.41] | | 0.81 |
| Age | 0.74 | 0.19 | 2.93 | [-5.01, 6.49] | | 0.80 |
| Newborn health risk | 0.06 | 0.17 | 0.14 | [-0.22, 0.34] | | 0.69 |
| **NC twin as reference** |  |  |  |  | |  |
| *Within-level covariates* |  |  |  |  | |  |
| Education | 0.99 | 0.27 | 2.20 | [-3.33, 5.31] | | 0.65 |
| Divorce | -1.30 | -0.36 | 1.19 | [-3.63, 1.04] | | 0.28 |
| Age | -0.67 | -0.09 | 2.48 | [-0.35, 0.25] | | 0.79 |
| Newborn health risk | -0.05 | -0.05 | 0.15 | [-0.44, 0.25] | | 0.74 |
| *Between level-covariates* |  |  |  |  | |  |
| Education | 0.57 | 0.42 | 2.22 | [-3.79, 4.93] | | 0.80 |
| Divorce | 0.48 | 0.45 | 1.30 | [-2.06, 3.03] | | 0.71 |
| Age | 0.73 | 0.18 | 2.68 | [-4.51, 5.99] | | 0.78 |
| Newborn health risk | 0.06 | 0.17 | 0.13 | [-0.20, 0.31] | | 0.67 |
|  | **Adolescent’s attachment avoidance to father** | | | | | |
|  | B | β | SE(B) | CI (B) | | p |
| **ART twin as reference** |  |  |  |  | |  |
| *Within-level covariates* |  |  |  |  | |  |
| Divorce | -3.85 | -0.45 | 2.11 | [-7.99, .28] | | 0.07 |
| Child age | 0.24 | 0.01 | 3.01 | [-5.67, 6.14] | | 0.94 |
| *Between level-covariates* |  |  |  |  | |  |
| Divorce | 2.34 | 0.55 | 2.49 | [-2.54, 7.21] | | 0.35 |
| Child age | -0.50 | -0.05 | 2.84 | [-6.06, 5.06] | | 0.86 |
| **ART singleton as reference** |  |  |  |  | |  |
| *Within-level covariates* |  |  |  |  | |  |
| Divorce | -3.85 | -0.45 | 2.11 | [-7.99, 0.28] | | 0.07 |
| Child age | 0.24 | 0.01 | 3.01 | [-5.67, 6.14] | | 0.94 |
| *Between level-covariates* |  |  |  |  | |  |
| Divorce | 2.33 | 0.55 | 2.49 | [-2.54, 7.21] | | 0.35 |
| Child age | -0.48 | -0.05 | 2.84 | [-6.05, 5.09] | | 0.87 |
| **NC twin as reference** |  |  |  |  | |  |
| *Within-level covariates* |  |  |  |  | |  |
| Divorce | -3.86 | -0.45 | 2.11 | [-7.99, .28] | | 0.07 |
| Child age | 0.24 | 0.01 | 3.01 | [-5.67, 6.14] | | 0.94 |
| *Between level-covariates* |  |  |  |  | |  |
| Divorce | 2.35 | 0.55 | 2.49 | [-2.53, 7.23] | | 0.35 |
| Child age | -0.51 | -0.05 | 2.84 | [-6.07, 5.05] | | 0.86 |
|  | **Adolescent’s attachment avoidance to best friend** | | | | | |
|  | B | β | SE(B) | CI (B) | | p |
| **ART twin as reference** |  |  |  |  | |  |
| *Within-level covariates* |  |  |  |  | |  |
| Sex | 2.71 | 0.40 | 3.07 | [-3.30, 8.72] | | 0.38 |
| Education | 3.09 | 0.45 | 2.00 | [-0.83, 7.01] | | 0.12 |
| Current dating | -1.58 | -0.23 | 6.18 | [-13.69, 10.53] | | 0.80 |
| *Between level-covariates* |  |  |  |  | |  |
| Sex | 0.64 | 0.25 | 3.36 | [-5.96, 7.23] | | 0.85 |
| Education | -1.00 | 0.48 | 2.02 | [-4.97, 2.96] | | 0.62 |
| Current dating | -0.05 | 0.04 | 6.79 | [-13.37, 13.26] | | 0.99 |
| **ART singleton as reference** |  |  |  |  | |  |
| *Within-level covariates* |  |  |  |  | |  |
| Sex | 2.71 | 0.40 | 4.59 | [-6.28, 11.70] | | 0.56 |
| Education | 3.10 | 0.45 | 2.00 | [-0.81, 7.02] | | 0.12 |
| Current dating | -1.57 | -0.23 | 10.37 | [-21.90, 18.76] | | 0.88 |
| *Between level-covariates* |  |  |  |  | |  |
| Sex | 0.64 | 0.25 | 5.07 | [-9.31, 10.58] | | 0.90 |
| Education | -1.02 | -0.49 | 2.01 | [-4.97, 2.93] | | 0.61 |
| Current dating | -0.07 | -0.05 | 11.45 | [-4.97, 2.93] | | 1.00 |
| **NC twin as reference** |  |  |  |  | |  |
| *Within-level covariates* |  |  |  |  | |  |
| Sex | 2.73 | 0.40 | 2.20 | [-1.59, 7.05] | | 0.22 |
| Education | 3.08 | 0.45 | 2.06 | [-0.96, 7.12] | | 0.14 |
| Current dating | -1.67 | -0.25 | 1.99 | [-5.56, 2.22] | | 0.40 |
| *Between level-covariates* |  |  |  |  | |  |
| Sex | 0.62 | 0.25 | 2.37 | [-4.03, 5.26] | | 0.80 |
| Education | -0.99 | -0.48 | 2.09 | [-5.08, 3.10] | | 0.64 |
| Current dating | 0.04 | 0.006 | 2.11 | [-4.09, 4.17] | | 0.99 |
|  | **Adolescent’s attachment anxiety to partner** | | | | | |
|  | B | β | SE(B) | CI (B) | p | |
| **ART twin as reference** |  |  |  |  |  | |
| *Within-level covariates* |  |  |  |  |  | |
| Education | 0.39 | 0.09 | 1.12 | [-1.81, 2.58] | 0.73 | |
| *Between-level covariates* |  |  |  |  |  | |
| Education | 0.75 | 0.69 | 1.14 | [-1.49, 2.99] | 0.51 | |
| **ART singleton as reference** |  |  |  |  |  | |
| *Within-level covariates* |  |  |  |  |  | |
| Education | 0.23 | 0.05 | 0.93 | [-1.60, 2.06] | 0.80 | |
| *Between-level covariates* |  |  |  |  |  | |
| Education | 0.92 | 0.82 | 0.96 | [-0.96, 2.81] | 0.34 | |
| **NC twin as reference** |  |  |  |  |  | |
| *Within-level covariates* |  |  |  |  |  | |
| Education | 0.34 | 0.08 | 1.00 | [-1.61, 2.29] | 0.74 | |
| *Between-level covariates* |  |  |  |  |  | |
| Education | 0.81 | 0.75 | 0.94 | [-1.04, 2.65] | 0.39 | |
|  | **Adolescent attachment avoidance to partner** | | | | | |
|  | B | β | SE(B) | CI (B) | p | |
| **ART twin as reference** |  |  |  |  |  | |
| *Within-level covariates* |  |  |  |  |  | |
| Child sex | 0.25 | 0.04 | 1.93 | [-3.54, 4.04] | 0.90 | |
| Education | 1.45 | 0.24 | 1.83 | [-2.13, 5.03] | 0.43 | |
| Current dating | -1.04 | -0.17 | 1.56 | [-4.11, 2.03] | 0.51 | |
| *Between-level covariates* |  |  |  |  |  | |
| Child sex | 0.62 | 0.39 | 2.00 | [-3.30, 4.53] | 0.76 | |
| Education | 0.98 | 0.69 | 1.96 | [-2.86, 4.81] | 0.62 | |
| Current dating | -1.21 | -0.73 | 1.59 | [-4.33, 1.91] | 0.45 | |
| **ART singleton as reference** |  |  |  |  |  | |
| *Within-level covariates* |  |  |  |  |  | |
| Child sex | 0.19 | 0.03 | 2.01 | [-3.74, 4.13] | 0.93 | |
| Education | 1.58 | 0.26 | 2.36 | [-3.06, 6.21] | 0.51 | |
| Current dating | -1.13 | -0.19 | 1.72 | [-4.50, 2.24] | 0.51 | |
| *Between-level covariates* |  |  |  |  |  | |
| Child sex | 0.68 | 0.47 | 2.10 | [-3.44, 4.80] | 0.75 | |
| Education | 0.82 | 0.53 | 2.54 | [-4.16, 5.80] | 0.75 | |
| Current dating | -1.12 | -0.63 | 1.78 | [-4.60, 2.37] | 0.53 | |
| **NC twin as reference** |  |  |  |  |  | |
| *Within-level covariates* |  |  |  |  |  | |
| Child sex | 0.23 | 0.04 | 17.13 | [-33.34, 33.79] | 0.99 | |
| Education | 1.60 | 0.26 | 40.62 | [-78.00, 81.20] | 0.97 | |
| Current dating | -1.06 | -0.18 | 27.18 | [-54.33, 52.22] | 0.97 | |
| *Between-level covariates* |  |  |  |  |  | |
| Child sex | 0.64 | 0.44 | 19.01 | [-36.62, 37.91] | 0.97 | |
| Education | 0.81 | 0.52 | 44.06 | [-85.55, 87, 16] | 0.99 | |
| Current dating | -1.20 | -0.65 | 28.63 | [-57.31, 54.91] | 0.97 | |

*Note.* ART = assisted reproductive treatment. NC = natural conception. SE = standard error. CI = confidence interval. In the models, the reference group (e.g., ART twin) is compared to the other three groups (e.g., ART singleton, NC twin, NC singleton) and covariates are used both in within- and between-levels for each model. The model for attachment anxiety to best friend included no covariates, so its results were not reported here.

| Supplementary Table III: *Main analyses results without covariates* | | | | | | | | | | |
| --- | --- | --- | --- | --- | --- | --- | --- | --- | --- | --- |
|  | Adolescent’s attachment anxiety to mother | | | | | Adolescent’s attachment avoidance to mother | | | | |
| *Contrasts* | B | β | SE(B) | CI (B) | *p* | B | β | SE(B) | CI (B) | *p* |
| ART twin vs. ART singleton | 0.53 | 0.21 | 0.33 | [-0.12, 1.18] | 0.11 | 0.48 | 0.06 | 1.43 | [-2.33, 3.29] | 0.76 |
| ART twin vs. NC twin | 1.33 | 0.14 | 0.85 | [-0.34, 3.00] | 0.12 | 1.22 | 0.05 | 1.61 | [-1.93, 4.38] | 0.45 |
| ART twin vs. NC singleton | 0.34 | 0.15 | 0.33 | [-0.31, 0.99] | 0.31 | 0.55 | 0.07 | 1.30 | [-2.01, 3.10] | 0.68 |
| ART singleton vs. NC twin | 0.78 | 0.09 | 0.79 | [-0.76, 2.32] | 0.32 | 0.68 | 0.03 | 2.14 | [-3.52, 4.88] | 0.75 |
| ART singleton vs. NC singleton | -0.22 | -0.08 | 0.26 | [-0.07, 0.29] | 0.41 | 0.02 | 0.009 | 0.77 | [-1.48, 1.52] | 0.98 |
| NC twin vs. NC singleton | -1.16 | -0.46 | 0.81 | [-2.75, 0.42] | 0.15 | -1.08 | -0.14 | 2.63 | [-6.24, 4.08] | 0.68 |
|  | Adolescent’s attachment anxiety to father | | | | | Adolescent’s attachment avoidance to father | | | | |
|  | B | β | SE | CI (B) | *p* | B | β | SE | CI (B) | *p* |
| ART twin vs. ART singleton | -0.89 | -0.54 | 0.50 | [-1.87, 0.10] | 0.08 | -1.41 | -0.19 | 1.46 | [-4.28, 1.45] | 0.33 |
| ART twin vs. NC twin | 1.67 | 0.30 | 0.93 | [-0.16, 3.50] | 0.07 | 3.68 | 0.14 | 2.95 | [-2.11, 9.46] | 0.21 |
| ART twin vs. NC singleton | -0.99 | -0.62 | 0.43 | [-1.83,-0.15] | **0.02** | 0.98 | 0.14 | 1.10 | [-1.17, 3.13] | 0.37 |
| ART singleton vs. NC twin | 2.52 | 0.41 | 0.97 | [0.62, 4.42] | **0.009** | 5.10 | 0.19 | 3.17 | [-0.08, 0.46] | 0.11 |
| ART singleton vs. NC singleton | -0.14 | -0.08 | 0.30 | [-0.73, 0.45] | 0.65 | 2.41 | 0.32 | 0.95 | [-0.03, 0.67] | **0.01** |
| NC twin vs. NC singleton | -2.71 | -1.50 | 0.88 | [-4.43, -.99] | **0.002** | -1.72 | -0.24 | 2.71 | [-7.03, 3.60] | 0.53 |
|  | Adolescent’s attachment anxiety to best friend | | | | | Adolescent’s attachment avoidance to best friend | | | | |
|  | B | β | SE | CI (B) | p | B | β | SE | CI (B) | p |
| ART twin vs. ART singleton | 0.27 | 0.09 | 0.77 | [-1.25, 1.78] | 0.73 | -1.14 | -0.31 | 0.89 | [-2.88, 0.60] | 0.54 |
| ART twin vs. NC twin | 0.37 | 0.04 | 1.009 | [-1.61, 2.35] | 0.71 | -0.06 | -0.04 | 1.70 | [-3.97, 2.70] | 0.71 |
| ART twin vs. NC singleton | -0.15 | -0.08 | 0.62 | [-1.36, 1.07] | 0.81 | -2.05 | -0.58 | 1.17 | [-4.35, 0.24] | 0.08 |
| ART singleton vs. NC twin | 0.07 | 0.02 | 0.90 | [-1.68, 1.83] | 0.94 | 0.42 | 0.05 | 1.77 | [-3.05, 3.88] | 0.81 |
| ART singleton vs. NC singleton | -0.44 | -0.16 | 0.45 | [-1.33, 0.45] | 0.33 | -0.93 | -0.32 | 0.71 | [-2.33, 0.46] | 0.19 |
| NS twin vs. NC singleton | -0.87 | -0.34 | 1.00 | [-2.83, 1.09] | 0.38 | -1.84 | -0.58 | 1.90 | [-2.46, 1.31] | 0.33 |
|  | Adolescent’s attachment anxiety to partner | | | | | Adolescent’s attachment avoidance to partner | | | | |
|  | B | β | SE | CI (B) | p | B | β | SE | CI (B) | p |
| ART twin vs. ART singleton | 0.99 | 0.49 | 0.51 | [-0.01, 1.99] | 0.053 | 0.64 | -0.30 | 0.92 | [-2.44, 1.16] | 0.49 |
| ART twin vs. NC twin | -0.83 | -0.12 | 1.09 | [-2.97, 1.32] | 0.45 | 1.61 | 0.20 | 1.43 | [-1.19, 4.41] | 0.26 |
| ART twin vs. NC singleton | 1.00 | 0.49 | 0.47 | [0.07, 1.93] | **0.04** | 0.01 | 0.008 | 0.99 | [-1.93, 1.96] | 0.99 |
| ART singleton vs. NC twin | -1.81 | -0.22 | 1.13 | [-4.03, 0.41] | 0.11 | 2.24 | 0.31 | 1.62 | [-0.47, 1.09] | 0.17 |
| ART singleton vs. NC singleton | 0.02 | -0.008 | 0.36 | [-0.69, 0.73] | 0.96 | 0.66 | 0.31 | 0.53 | [-0.64, 1.25] | 0.21 |
| NC twin vs. NC singleton | 1.63 | 0.71 | 1.11 | [-0.53, 3.80] | 0.14 | -1.24 | -0.51 | 1.64 | [-4.46, 1.97] | 0.45 |

*Note.* ART = assisted reproductive treatment. NC = natural conception. SE = standard error. CI = confidence interval. Model for attachment anxiety to best friend included no covariates, so its results are the same as in Table V in the main results. Statistically significant p-values (p < 0.05) are bolded.
